# Neurotoxic tau oligomers after single versus repetitive mild traumatic brain injury

**DOI:** 10.1093/braincomms/fcz004

**Published:** 2019-06-28

**Authors:** Alice Bittar, Nemil Bhatt, Tasneem F Hasan, Mauro Montalbano, Nicha Puangmalai, Salome McAllen, Anna Ellsworth, Mariana Carretero Murillo, Giulio Taglialatela, Brandon Lucke-Wold, Aric Logsdon, Charles Rosen, Ryan C Turner, Rakez Kayed

**Affiliations:** 1 Department of Neurology, The Mitchell Center for Neurodegenerative Diseases, University of Texas Medical Branch, Galveston, TX 77555-1045, USA; 2 Department of Neurology, Ochsner Louisiana State University Health Sciences Center, Shreveport, LA 71103, USA; 3 Baylor College of Medicine, Houston, TX 77030, USA; 4 Department of Neurosurgery, University of Florida, Gainesville, FL 32608, USA; 5 Department of Psychiatry, University of Washington, Seattle, WA 98195, USA; 6 Central Illinois Neural Health Sciences, Bloomington, IL 61701, USA; 7 Department of Neurosurgery, Health Sciences Center, West Virginia University School of Medicine, Morgantown, WV 26506, USA

**Keywords:** traumatic brain injury, tau oligomers, tau polymorphisms, tau strains, neurodegeneration

## Abstract

Mild traumatic brain injury accounts for the majority of head injuries and has been correlated with neurodegeneration and dementia. While repetitive mild traumatic brain injury is highly correlated to neurodegeneration, the correlation of a single mild traumatic brain injury with neurodegeneration is still unclear. Because tau aggregates are the main form of mild traumatic brain injury induced pathology, toxic forms of tau protein most likely play a role in the development of post-mild traumatic brain injury neurodegeneration. Therefore, it becomes crucial to characterize the properties of soluble tau aggregates in single versus repetitive mild traumatic brain injury. Herein, we isolated tau oligomers from wild-type mice exposed to single or repetitive mild traumatic brain injury and characterized the tau aggregates at functional, biochemical and biophysical levels. We demonstrated that single versus repetitive mild traumatic brain injuries frequencies lead to the formation of different tau oligomeric polymorphisms. These polymorphisms express different long-term potentiation impairment potencies, toxicity potentials, morphologies and strain indicating properties. To our knowledge, this is the first evidence that soluble tau oligomers derived from single versus repetitive mild traumatic brain injuries form distinct polymorphisms that possibly correlate with the risk of neurodegeneration after mild traumatic brain injury.

## Teaser

Soluble tau aggregates have been highlighted in mTBI-induced cellular dysfunction and neurodegeneration. We used an established mTBI model to investigate differences between tau oligomers isolated after single vs. repetitive mTBI. Single and repeated blast tau oligomers expressed distinct strain-indicating characteristics that correlated with the risk of neurodegeneration.

## Graphical Abstract

**Graphical Abstract:**
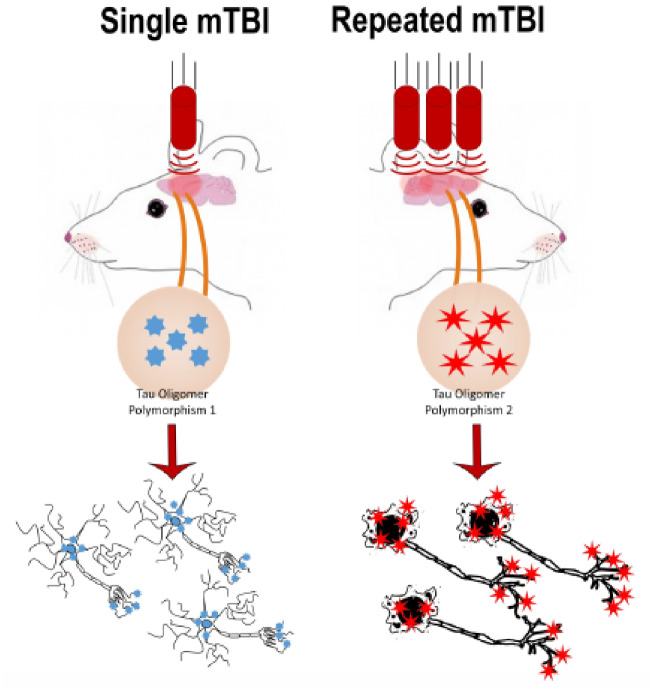

## Introduction

The association between traumatic brain injury (TBI) and neurodegeneration is not new. Mild TBI (mTBI) is now widely accepted as one of the highest risks and greatest epigenetic factors for Alzheimer’s disease and several other neurodegenerative tauopathies (Chauhan, 2014; Faden and Loane, 2015; Washington *et al.*, 2016). More than 80% of the annually reported/hospitalized TBIs among civilians are mild (Cassidy *et al.*, 2004). In addition, most of the injuries reported by military personnel or contact sports athletes are also mild (Langlois *et al.*, 2006; Vanderploeg *et al.*, 2012). mTBI patients score >13 on the standard Glasgow Coma scale (Teasdale and Jennett, 1974), and brain damage due to an mTBI is not necessarily detectable by the neuroimaging techniques available in hospitals (Blyth and Bazarian, 2010). Therefore, diagnosing mTBI is tricky and dependent on clinical exam. Moreover, according to the American Academy of Neurology ‘back to play’ guidelines, veterans often return to the battlefield and athletes to their games 1 week after being asymptomatic (Blyth and Bazarian, 2010). Those guidelines are heavily based on clinical observations that do not take into consideration neurodegeneration-inducing events at the protein level.

While clinical studies fail to show a correlation between single mTBI and neurodegeneration, a single mTBI may render the brain more sensitive to further traumatic brain injuries and long-term functional impairments (Blyth and Bazarian, 2010). Repetitive mTBI, on the other hand, is strongly correlated with neurodegeneration and dementia. American football athletes and military personnel with a history of multiple mild concussions show neurodegeneration, cognitive deficits and tau-related pathology. Additionally, they show a correlation with a broad spectrum of neurodegenerative and psychiatric diseases (McKee *et al.*, 2013; Aungst *et al.*, 2014; Baugh *et al.*, 2014; Luo *et al.*, 2017) including Alzheimer’s disease (Breunig *et al.*, 2013; Washington *et al.*, 2016), Parkinson’s disease (Acosta *et al.*, 2015; LoBue *et al.*, 2016) and Frontotemporal dementia (LoBue *et al.*, 2016). However, what makes repetitive mTBI so detrimental in comparison to single mTBI remains to be elucidated.

Extensive research shows that tau oligomers are causally linked to neurodegeneration and memory impairment post-mTBI (Lasagna-Reeves *et al.*, 2011; Gerson *et al.*, 2016; Kanaan *et al.*, 2016). Tau oligomers have been also shown to be involved in synaptic dysfunction, plasticity, pruning and the consequent chronic neuronal loss (Spires-Jones and Hyman, 2014; Fá *et al.*, 2016; Yin *et al.*, 2016). Hence, given the above, and keeping in mind that tau oligomers are the true toxic tau species (Kanaan *et al.*, 2016), it becomes of utmost importance to closely examine tau oligomers after single and repetitive mTBI in terms of their corresponding link to neurodegeneration and memory impairment.

This study utilizes a blast injury mouse model of TBI to explore and characterize tau oligomers in mouse brains exposed to single and repetitive mTBI. In brief, we compared tau oligomers immunoprecipitated from mouse brains exposed to single and repetitive mTBI using an array of well-established biochemical and biophysical techniques. Here, we report that tau oligomers that form after single and repetitive mTBI have distinct characteristics. The differences observed here, appear to be fundamental to their function and furthermore, to their categorization as different strains.

## Materials and methods

### Animals

All experimental protocols and animal handling procedures were performed in accordance with the ARRIVE guidelines and the National Institutes of Health (NIH) guidelines for the use of experimental animals and approved by the Institutional Animal Care and Use Committee.

### mTBI paradigm and isolation of soluble tau (tau oligomers) from mouse brains exposed to single and repetitive mTBI

**Figure 1:**
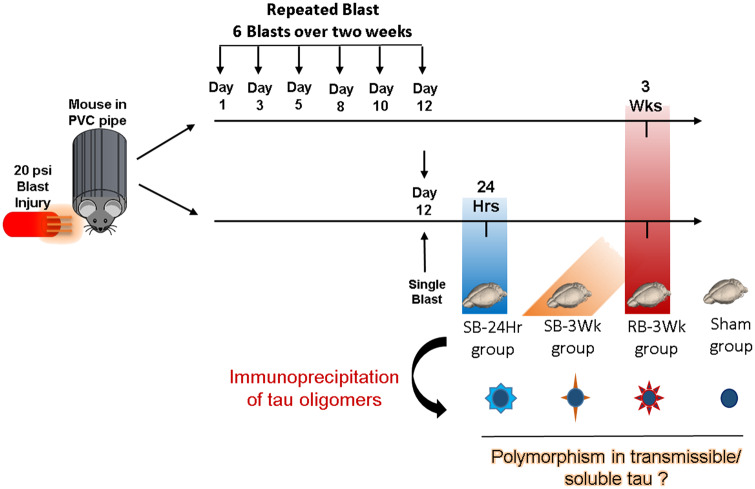
**TBI paradigm and timeline used in this study: The mice were placed in a PVC pipe and exposed to a 20 psi (mild to moderate) blast injury.** The mice were divided into four groups depending on the frequency of TBI and the time after TBI their brain was collected. The groups are: (i) SB-24Hr: brain isolated 24 h after the single blast, (ii) SB-3Wk: brain isolated 3 weeks after the single blast, (iii) RB-3Wk: brain isolated 3 weeks after the repeated blast, (iv) SHAM: brain isolated 3 weeks after sham TBI. Following brain isolation, soluble small aggregates (tau oligomers) were isolated by immunoprecipitation using oligomer-specific T22 antibody, followed by FPLC. Details about the immunoprecipitation protocol are explained in detail in Lasagna-Reeves *et al.* (2010).

Frozen mouse brain tissues were obtained from West Virginia University. The mice, from which the brains were obtained, were 6-month-old wild-type C57BL/6 male mice that had undergone blast-induced traumatic brain injury (mTBI) as described in Fig. 1 (Logsdon *et al.*, 2017). The mice were placed in a PVC pipe with the head extending from one side. A 20 psi blast wave was directed at the head to produce a mTBI. According to the ARRIVE guidelines, a control group, which is the sham-treated mice group was added.

A thorough characterization of the used blast model was previously performed on mice and rats (Turner *et al.*, 2013; Logsdon *et al.*, 2017). In brief, it was shown that a mild blast (20 psi in mice) caused disruption of vascular integrity and the blood–brain barrier (BBB) in the cortex starting at 72 h post-blast. The blast also caused an increase in astrocyte reactivity and glial fibrillary acidic protein expression in the cortex, as well as in oxidative stress markers and oligomeric tau levels (reactive to T22). A previous publication (Turner *et al.*, 2015) described tissue and axonal damage, however, in rats. In this publication, they observed intracranial bleeds, neural injury and swollen axons. In addition, they also observed neural degeneration and an increase in several inflammation markers in a blast dose-dependent manner starting at 72 h after injury in the cortex, hippocampus, corpus callosum and cerebellum. In addition to the clinically relevant histological and behavioural outcomes observed, this model is also relevant from the aspect of the peak overpressure blast wave given.

At the level of tau pathology, a thorough characterization was performed on mice and/or rats after single or repeated blasts. In brief, histological analysis was performed at 3 weeks after a single blast, and 1 month after repeated blast. Results showed a marked increase in phosphorylated tau pathology (AT8) and tau conformational change marker (CP13) in both groups. This increase in phosphorylated tau and tau conformational change markers temporally overlapped with the induction of memory and cognitive deficits in both single and repeated blast groups (Turner *et al.*, 2015; Logsdon *et al.*, 2017).

Based on the previous data mentioned above, the time points after blast for this study were chosen as follows: (i) 24 h after single blast (SB-24Hr), (ii) 3 weeks after single blast (SB-3Wk), (iii) 3 weeks after repetitive blast (RB-3Wk), (iv) 3 weeks after sham treatment (Sham). Sham mice were placed under the same conditions as the other groups, but with no blast. The repetitive blast was administered at an interval of six blasts over the course of 12 days.

Then, mouse brains were harvested right after euthanasia at each of the mentioned time points according to the ARRIVE guidelines and tau oligomers were immunoprecipitated from the prefrontal cortex region. The brains from each experimental group were coded with numbers to keep the experimenter blind from the blast received by each brain. Immunoprecipitated oligomers from 14 brains that received single and repetitive blasts were obtained. Four oligomeric samples were chosen from the 14 for further analysis in this study. The choice was made based on the concentration, molecular weights and the PK Digestion profiles of the oligomeric isolates. The immunoprecipitation was performed using T22, an oligomer-specific antibody, followed by fast protein-lipid chromatography (FPLC) using our previously published protocols (Lasagna-Reeves *et al.*, 2010). Tau oligomers were characterized according to well-established functional, biochemical and biophysical methods, thus forming a novel sequence of tests that can be used to characterize and compare brain-derived tau oligomers.

In brief, the immunoprecipitation of tau oligomers from mTBI mouse brains was completed as previously described (Lasagna-Reeves *et al.*, 2012*a*; Sengupta *et al.*, 2018). Thirty microlitres of tosyl-activated magnetic Dynabeads (Dynal Biotech, Lafayette Hill, PA, USA) were coated with 20 μg of anti-tau oligomer-specific polyclonal antibody, T22 (1.0 mg/ml) and diluted in 50 μl of 0.1 M borate, pH 9.5, thus keeping end concentration of beads at 20 mg/ml. The beads were kept overnight at 37°C. The beads then were washed (0.2 M Tris-HCl, 0.1% bovine serum albumin, pH 8.5) and incubated with 100 μl of the mTBI brain homogenates [phosphate-buffered saline (PBS) soluble fraction] and rotated for 1 h at room temperature. The beads were then washed three times with PBS and eluted using 0.1 M glycine at pH 2.8. Fractions were centrifuged in an amicrocon centrifugal filter device. The molecular weight cut-off was 25 kDa (Millipore, Cat # 42415) at 14 000 g for 25 min at 4°C. Oligomers were re-suspended in sterile PBS and protein concentration was measured using the bicinchoninic acid protein assay (Pierce). The samples were centrifuged again in an amicrocon centrifugal filter device (cut-off 25 kDa) at 14 000 g for 25 min at 4°C. Oligomers were stored at −80°C until use (Lasagna-Reeves *et al.*, 2012*a*). Oligomers characterization followed the previously established systematic approach described in Lasagna-Reeves *et al.* (2010).

### Oligomer amplification

Recombinant tau 4R monomers that were obtained by dissolving lyophilized pellets of recombinant 4R tau at 1 mg/ml concentration in PBS (Lasagna-Reeves *et al.*, 2011) were seeded with mTBI brain-derived tau oligomers. The oligomer-monomer mixture was made at a ratio of 1:100 (w/w) with rotation at room temperature for 4 h. Aliquots were taken and immediately used for western blotting and atomic force microscopy (AFM) analysis each time seeding was done to ensure quality maintenance.

### Immunodepletion of tau oligomers

The tau oligomeric samples were pre-incubated with T22 and TOMA1 antibodies at a ratio of 1:4 (Oligomer to antibody). The oligomer-antibody complexes were rotated for 2 h at room temperature before treating the cells. The same method of pre-incubation was used for the toxicity assays and the FRET cell staining.

### Western blot analysis

Ten micrograms of each sample were loaded in a precast SDS-PAGE Invitrogen (NuPAGE 4–12% Bis-Tris) gel and transferred to nitrocellulose membranes. Membranes then were blocked with 10% non-fat milk in tween-Tris-buffered saline with (TBS-T, tween 0.01%) overnight at 4°C. The following day, membranes were probed with T22 (1:250) for tau oligomers, TOMA 1 (1:250) and Tau 5 (1:10 000) for total tau, diluted in 5% non-fat milk for 1 h at room temperature. Membranes were then incubated with horseradish peroxidase-conjugated IgG anti-rabbit (1:10 000) and anti-mouse (1:10 000) secondary antibodies to detect, T22, TOMA1 and Tau 5, respectively. ECL plus (GE Healthcare) was used for signal detection.

### Filter trap assay

Filter trap assay was performed to detect N and C termini of tau oligomers with conformational antibodies in the absence of reducing agents, as we have shown earlier (Ghag *et al.*, 2018; Lo Cascio and Kayed, 2018). In brief, TBI brain-derived tau oligomers (1 μg) were applied to a nitrocellulose membrane that was saturated with TBS-T via a vacuum-based bio-slot apparatus. Membranes were then blocked with 10% non-fat milk at 4°C for 24 h. Membranes were then probed with the C-terminal tau antibody Tau46 (1:18 000) and the C-terminal tau antibody Tau13 (1:50 000) diluted in 5% non-fat milk for 1 h at room temperature. Membranes were then washed three times with TBS-T and incubated with horse radish peroxidase-conjugated anti-mouse IgG (1:6000, GE Healthcare). Membranes were washed again three times in TBS-T and ECL plus (GE Healthcare) was used for signal detection.

### MTT and lactate dehydrogenase assays

Human neuroblastoma SH-SY5Y cells and cortical primary neurons obtained from wild-type C57/B6 embryos were maintained in their appropriate cell culture medium and grown to around 60% confluency in 96-well plates. SH-SY5Y cells were treated for 24 h with 0.125, 0.25 and 0.5 μM of tau oligomers. Primary neurons were treated for 24 h with 0.5, 1 and 2 μM of tau oligomers. Cell viability was determined using 3-(4,5-dimethylthiazol-2-yl)-2,5-diphenyltetrazolium bromide (MTT) assay. The cytotoxic effect was determined using the lactate dehydrogenase (LDH) assay for assessing cell cytotoxicity following manufacturers’ instructions. Optical density (OD) was measured at 490 nm with a POLARstar OMEGA plate reader (BMG Labtechnologies). Cell viability was calculated as the percentage of the OD value of treated cells compared with untreated controls, according to the following equation: Viability = (OD SAMPLE/OD CONTROL) × 100%, after correcting for the vehicle-alone background. Cellular cytotoxicity was calculated as the percentage of OD value of treated cells minus low control which is untreated cells compared with high control which is maximum cytotoxicity according to the following formula: Cytotoxicity = (OD Sample-low control)/(High control-Low control) × 100%. All measurements were performed in triplicates. Statistical analysis was based on a two-way analysis of variance (ANOVA) followed by Bonferroni multiple comparisons test.

### Atomic force microscopy

Tau oligomers were characterized by AFM as previously described (Lasagna-Reeves *et al.*, 2010). In brief, samples were prepared by adding 10 μl of tau oligomers on freshly cleaved mica. The mica was left at room temperature for 1 day allowing the oligomers to adsorb to the surface. Mica was then washed three times with distilled water to remove unbound protein and impurities followed by air-drying. Samples were then imaged with a Multimode 8 AFM machine (Veeco, CA) using a noncontact tapping method (ScanAsyst-Air).

### Proteinase K digestion

To evaluate differences in stability between tau oligomers from single and repetitive mTBI, Proteinase K (PK) enzyme was used to treat tau oligomer samples at a ratio of 0–1 μg/ml of enzyme in water. Tris-HCl and NaCl were added to each sample and PK enzyme mixture so that the final concentrations of Tris-HCl and NaCl were 150 and 100 mM, respectively. Samples were treated with PK for 1 h at 37°C. The reaction of the enzyme was stopped by adding 4× SDS-PAGE loading buffer (NuPAGE LDS sample buffer, Invitrogen). Samples were then incubated at 95°C for 5 min. The total reaction volume for each sample was immediately used for analysis by western blot.

### Electrophysiology

Acute brain slices methods are described in Comerota *et al.* (2017). In brief, mice (*n* = 6) were deeply anaesthetized with isoflurane and transcardially perfused with 25–30 ml of carbogenated (95% O_2_ and 5% CO_2_ gas mixture) N-methyl-D-gluconate-artificial cerebrospinal fluid at room temperature. Transverse brain sections of 350 μm containing Schaffer collateral synapses were generated using the Compresstome VF-300 (Precisionary Instruments, Greenville, NC, USA). Slices were allowed to recover in NMDG-HEPES cutting solution at 32–34°C for 10 min and were then transferred to carbogen bubbling HEPES-artificial cerebrospinal fluid solution at room temperature for the rest of the experiment time. Slices were treated with oligomers after the 10 min recovery where they were transferred to separate chambers and incubated with 50 nM of each oligomeric sample diluted in carbogen bubbling HEPES-artificial cerebrospinal fluid solution at room temperature for 1 h before recording. After treatment, the slices were briefly (5–10 min) washed by placing them in an oligomer-free recovery artificial cerebrospinal fluid before placing them on the recording stage. Two slices at a time were transferred to the recording chamber and perfused with carbogen bubbling room temperature normal artificial cerebrospinal fluid at a rate of approximately 3 ml/min for recordings. Recording electrodes were pulled from borosilicate glass capillaries using a horizontal P-97 Flaming/Brown micropipette puller (Sutter Instruments, Novato, CA). Evoked field excitatory post-synaptic potentials (fEPSPs) in the CA1 were obtained by stimulating Schaffer collateral. A stable baseline was obtained by delivering single-pulse stimulation at 20-s interstimulus intervals. Lhong-term potentiation (LTP) was induced by exposing the slices to high-frequency stimulation (HFS; 3 × 100 Hz, 20 s) using Digidata 1550B (Molecular Devices, Sunnyvale, CA, USA), and measured using an Axon MultiClamp 700B differential amplifier (Molecular Devices). Clampex 10.6 software (Molecular Devices) was used. All data are represented as percent change from the initial average baseline fEPSP slope, which was defined as the average slope obtained for 10 min before HFS.

### Synaptosomes fractionation

Isolation of synaptosomes containing the pre- and post-synaptic markers was done using the Syn-PER reagent (Thermo Fisher Scientific, Waltham, MA, USA) following the manufacturer’s protocol. Whole intact hippocampal tissue was isolated from 6-month-old C57/B6 mice and incubated with each of the oligomeric samples in hand for 1 h under aeration in carbogenated ACSF. Following incubation, the tissue was homogenized in Syn-PER reagent (1 ml Syn-PER/100mg of tissue) plus protease and phosphatase inhibitors. Using the dounce, the tissue was stroked 10 times and the suspension was centrifuged for 10 min at 1200× g. The supernatant fraction was collected and then further centrifuged at 15 000× g for 20 min. Centrifugation took place at 4°C. The subsequent pellet which contains the wanted synaptosomes was re-suspended in HEPES-buffered Krebs-like buffer for western blot analysis.

### Seeding assay

Tau RD P301S biosensor cells were plated on coverslips in a 24 well plate (25 000 cells per well) and treated with each of the oligomeric samples (SB-24Hr, SB-3Wk, RB-3Wk and Sham) for 48 h at a concentration of 0.5 µM. The transduction medium was composed of Opti-MEM (Gibco) + Lipofectamine + oligomeric seeds (Holmes *et al.*, 2014; Furman *et al.*, 2015; Kaufman *et al.*, 2016). The untreated control media was composed of the Opti-MEM media + Lipofectamine. Cells were then fixed and imaged using confocal microscopy (Zeiss LSM 880).

### Statistical analysis

Data analysis was performed using Prism 7.0 software (GraphPad). One-way ANOVA was used to compare multiple groups with either Bonferroni or Sidak *post hoc* test, as indicated in each Figure’s legend. *P*-values less than 0.05 were considered statistically significant.

### Data availability

The authors confirm that all the data supporting the findings of this study are available within the article and its Supplementary material. Raw data will be shared by the corresponding author upon request.

## Results

### Biochemical characterization of the tau oligomers isolated from single versus repetitive mTBI-exposed brains

**Figure 2:**
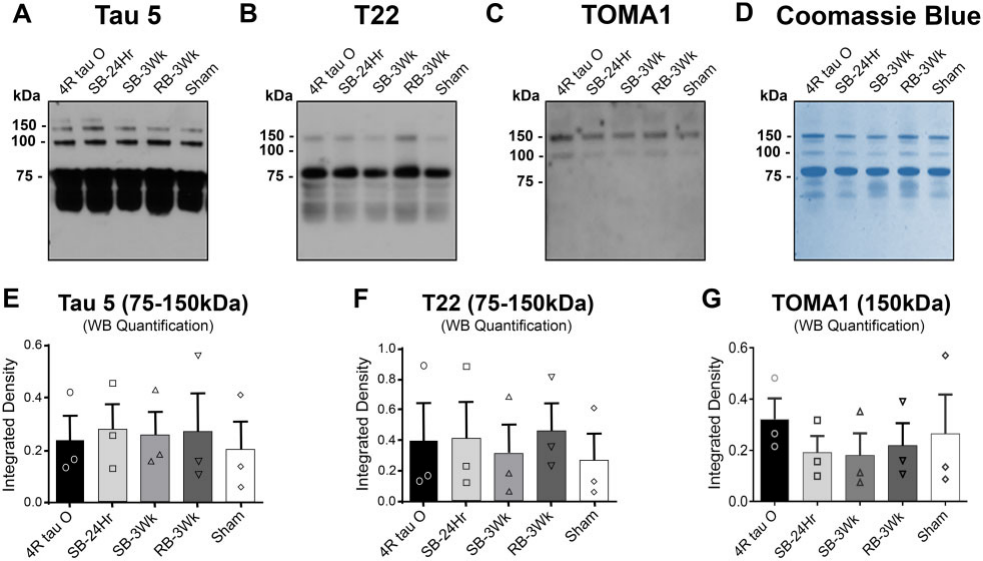
**Biochemical characterization of tau oligomers derived from brains exposed to single and repetitive TBI.** (**A–C**) Representative western blots of the tau oligomers probed with total tau antibody Tau5, polyclonal oligomer-specific antibody T22 and monoclonal oligomer-specific antibody TOMA1. Results show that Tau5 detects 75, 100 and 150 kDa molecular weight oligomers, T22 detects 75 and 150 kDa molecular weight oligomers and TOMA1 detects 100 and 150 kDa molecular weight oligomers. (**D**) Coomassie blue staining of the tau oligomers showing bands at high molecular weights (75–150 kDa) representing oligomers. (**E–G**) Western blot quantifications showing that the higher molecular aggregates were detected by all the tau-specific antibodies used, and that there are no significant differences in the immunoreactivity of each antibody with each of the samples. (Each treatment is compared with Sham; one-way ANOVA followed by Bonferroni *post hoc* test).

Tau oligomers were immunoprecipitated from mouse brains at (i) 24 h after single blast (SB-24Hr), (ii) 3 weeks after single blast (SB-3Wk), (iii) 3 weeks after repetitive blast (RB-3Wk), (iv) 3 weeks after sham treatment (Sham). The immunoprecipitated TBI brain-derived tau oligomers were amplified using tau4R monomer. To evaluate the oligomeric content of the immunoprecipitated samples, we used western blot analysis. The membranes were probed by three different tau-specific antibodies; Tau 5, a total tau antibody; T22, a polyclonal tau oligomer-specific antibody; and TOMA1, a monoclonal tau oligomer-specific (Fig. 2A–C). The bands between 75 and 150 kDa, which correspond to tau oligomers were used for quantification (Lasagna-Reeves *et al.*, 2010, 2012*b*) (Fig. 2E–G). To further evaluate the oligomeric content of the immunoprecipitated samples, we stained the membrane with Coomassie blue (Fig. 2D). The samples show bands between 75 and 150 kDa that correspond to tau oligomers. The samples were also run on a filter trap assay under nondenaturing conditions and probed with the C-terminal Tau46 and N-terminal antibodies Tau13 antibodies to confirm that the oligomeric samples represent full-length tau conformations (Supplementary Fig. 1C). Moreover, Supplementary Fig. 1D also shows an additional size characterization of the oligomeric samples. The FPLC chromatogram shows that the oligomeric samples express different size distribution profiles. SB-24Hr, SB-3Wk and the sham oligomers showed main peaks between 75 and 150 kDa that correspond to dimers and trimers, and additionally a small monomer-corresponding peak. The main peaks in RB-3Wk correspond to trimers and above (150–250 kDa). All experiments were repeated in triplicates for statistical significance, and representative western blots are shown in Fig. 2. (Each group was compared with the sham group using one-way ANOVA followed by Bonferroni *post hoc* test.)

### RB-3Wk oligomers are the most toxic among the mTBI brain-derived tau oligomers *in vitro*

**Figure 3:**
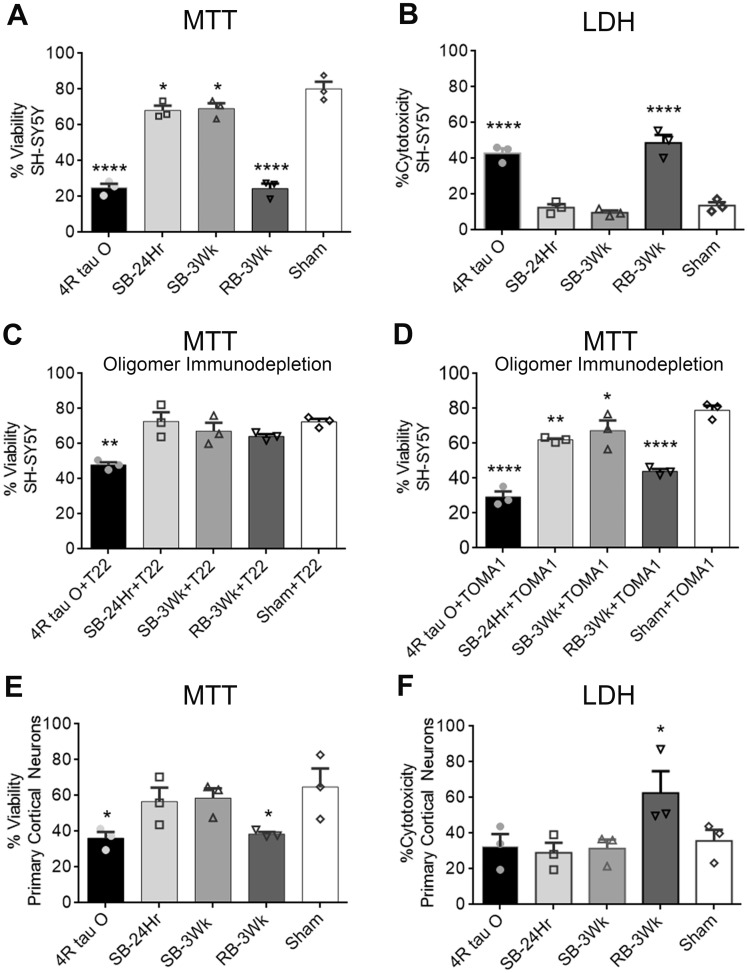
**Tau oligomers derived from brains exposed to single versus repetitive TBI express differential cellular toxicity and oligomer-specific antibodies rescue oligomer-induced toxicity.** SH-SY5Y cells and cortical primary neuronal cells (C57BL/6) were exposed to 0.5 µM concentration of the mTBI brain-derived tau oligomers. (**A**, **B**) Results showed that the oligomers caused differential percent viability and cytotoxicity, respectively, with the repetitive blast sample being the most toxic in SH-SY5Y cell culture in comparison to sham oligomers (**A**: *P* = 0.003, *F* (4, 10) = 8.493; **B**: ****P < 0.0001, *F* (4, 10) = 33.36). (**C**) T22 antibody pre-incubated with the oligomers at a ratio (1:4; oligomer to antibody) rescued the cellular toxicity caused by the repeated blast oligomers and 4R tau O (****P < 0.0001; *F* (2, 6) = 70.01). (**D**) Oligomer immunodepletion was also performed with TOMA1 antibody (**P = 0.0014; *F* (2, 6) = 23.55). Differential toxicity was still observed in comparison to the sham group. (**E**, **F**) MTT and LDH assays were also performed on primary cortical neuronal cultures. Results show that RB-3Wk oligomers were significantly higher in toxicity in comparison to sham and the single blast oligomers (*P = 0.0054, *F* (4, 10) = 7.187) (*P = 0.005, *F* (3, 8) = 19.90). One-way ANOVA followed by *post hoc* comparison, adjusted using Sidak correction. Results are expressed as a percent of untreated controls. Each treatment is compared with treatment with sham oligomers. Each assay was repeated three times).

To evaluate the toxicity of the oligomers isolated from single versus repetitive TBI-exposed brains *in vitro*, we ran LDH and MTT assays on SH-SY5Y cells and cortical primary neuronal cultures (from C57BL/6 wild-type mice) treated with the oligomers at a concentration of 0.5 µM. Figure 3A shows that all oligomeric groups significantly affected cellular viability in comparison to sham with the repeated blast oligomers being the highest in effect. The same trend was observed in Fig. 3B where only RB-3Wk and the recombinant tau 4R showed significant % cytotoxicity in comparison to sham oligomers. The SB-24Hr and SB-3Wk oligomers were comparable to the sham oligomers in % cytotoxicity (<20%) (one-way ANOVA followed by *post hoc* comparison adjusted for Sidak correction). To confirm that this toxicity effect is tau-mediated, we pre-incubated each of the five TBI brain-derived tau oligomers with T22 and TOMA1 (Fig. 3C and D). Percent viability significantly increased when comparing the RB-3Wk oligomers alone treatment with the ones pre-incubated with T22. This indicates that the toxicity seen is mediated by tau oligomers. This also indicates that T22 and TOMA1 antibodies were able to rescue and partially rescue, respectively, the cells from oligomer toxicity. No increase in cellular viability could be detected with the SB-24Hr and SB-3Wk oligomers pre-incubated with T22 and TOMA1. That is most likely because of the initial high percentage of viability obtained with the oligomer treatment only. The toxicity and pre-incubation experiments were run in parallel in triplicates for each treatment on the same 96-well plate for all the groups. The data of oligomer toxicity and pre-incubation are graphed separately for simpler data representation.

Similar experiments were repeated on primary neuronal cultures (Fig. 3E and F). A comparable trend of cellular toxicity was observed with the primary neuronal cultures. Only the RB-3Wks oligomers were significantly toxic in comparison to sham oligomers (Fig. 3E and F).

### Single versus repetitive mTBI cause the formation of functionally different tau oligomers

Brain-derived tau oligomers have been shown to affect neuronal transmission and impair hippocampal LTP which reflects memory *in vivo* (Lasagna-Reeves *et al.*, 2011; Fá *et al.*, 2016), one of the main functions affected by TBI. Hence, we further characterized the mTBI brain-derived oligomers by testing their effects on basal neuronal transmission, paired-pulse facilitation and LTP. Representative traces before (black) and after (red) HFS of each group are shown above the LTP impairment graph.

**Figure 4:**
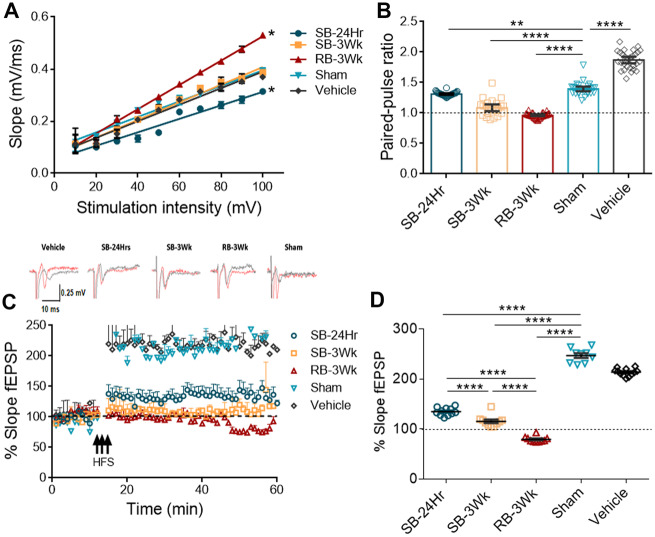
**Tau oligomers derived from brains exposed to single versus repetitive TBI impair LTP to different magnitudes with the sample from the repetitive blast showing the most impairment.** Hippocampal slices (350 µm thickness) were treated with the oligomers for 1 h at 50nM. (**A**) The input–output curve shows the RB-3Wk oligomers increase basal neuronal transmission (*R*^2^ = 0.9922, *P < 0.0001) whereas the SB-24Hr oligomers decrease it (*R*^2^ = 0.9484, *P < 0.0001) in comparison to vehicle control (*R*^2^ = 0.9743). (**B**) Paired-pulse ratios results show that all the oligomers impaired facilitation in comparison to sham-treated groups, and RB-3Wk oligomers completely abolished it. (**C**) LTP impairment profiles show that RB-3Wk and SB-3Wk abolish LTP whereas SB-24Hr partially impairs LTP in comparison to sham oligomers. Representative traces before (black) and after (red) LTP are shown above LTP plot. (**D**) The last 10 min of LTP were analysed separately. Results show that LTP is significantly impaired with the single blast oligomers. The repetitive blast oligomers show a synaptic depression effect. Linear regression analysis indicating significantly different slopes from vehicle treatment in **A** (****P < 0.00001, **P < 0.001). (one-way ANOVA was used in **B** and **D**; *post hoc* comparison: adjusted using Sidak correction. *n* = 6 slices).

Since basal neuronal transmission increases after mTBI (Carron *et al.*, 2016), the effect of mTBI brain-derived tau oligomers on basal neuronal transmission was tested (Fig. 4A). Input–output curves show that basal neuronal transmission significantly increases (*R*^2^ = 0.9922) after incubation with the RB-3Wk oligomers as seen by the significantly increased slope of the curve when compared with vehicle. On the other hand, SB-24Hr oligomers decrease basal neuronal transmission (*R*^2^ = 0.9484) evident by the decreased slope in comparison to the vehicle (*R*^2^ = 0.9743). Oligomers from SB-3Wks group did not significantly affect basal neuronal transmission when compared with the vehicle-treated or sham oligomers-treated groups (Fig. 4A, *n* = 6 slices). In addition to basal neuronal transmission, paired-pulse ratio significantly decreased after treatment with SB-24Hr, SB-3Wk and RB-3Wk groups of oligomers in comparison to sham and vehicle treatment. However, paired-pulse facilitation was completely abolished only after treatment with the RB-3Wk group (Fig. 4B, *n* = 6 slices). Last but not least, Fig. 4C shows that tau oligomers of SB-24Hr, SB-3Wk and RB-3Wk impair LTP in comparison to sham oligomers and the vehicle, but in different magnitudes. Representative traces before (black) and after (red) HFS of each treatment are shown above the LTP impairment graph. To better evaluate and obtain quantitative measures of the observed differences in the effect of the oligomers on LTP, fEPSP measurements from the last 10 min of LTP were analysed separately. Results in Fig. 4D show that, in comparison to sham, SB-24Hr, SB-3Wk and RB-3Wk oligomers impair LTP. However, RB-3Wk oligomers seem to decrease the fEPSP slope below the baseline possibly indicating a synaptic depression phenotype (*n* = 6 slices).

### Tau oligomers derived from brains exposed to single versus repetitive TBI differentially affect the expression levels of synaptic proteins crucial for synaptic transmission

**Figure 5:**
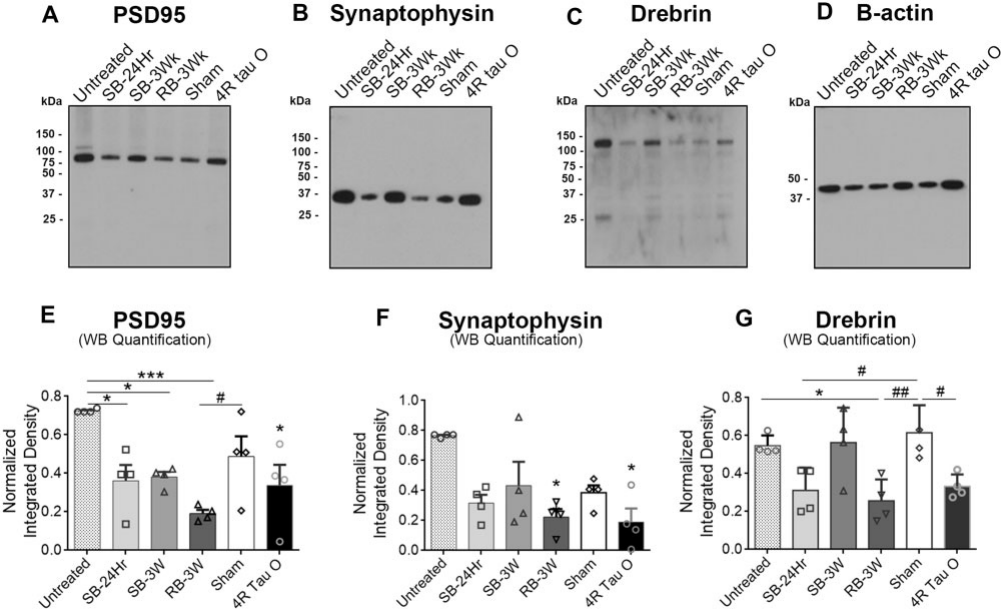
**Decrease in synaptic markers levels after incubation with TBI brain-derived tau oligomers from brains exposed to single versus repetitive TBI.** Fresh intact hippocampi from wild-type mice were incubated with the oligomers for 1 h. Synaptosomes were then isolated and run on a western blot probing for (**A**) PSD95, (**B**) Synaptophysin and (**C**) Drebrin. (**D**) β-actin blot. (**E**) Results show that PSD95 levels significantly decrease with the treatment with all the oligomers except the sham when compared with untreated control (***P < 0.0001, *F* (5, 12) = 6.660). Only RB-3Wk treatment decreased PSD95 levels in comparison to sham (*P = 0.0034, *F* (5, 12) =6.660). (**F**) Synaptophysin levels significantly decrease after treatment with RB-3W and the 4R tau oligomers when compared with untreated control (*P = 0.003, *F* (5, 12) = 11.28). (G) Drebrin levels significantly decrease only with the RB-3Wk oligomer treatment when compared with untreated control and to sham (**P = 0.0025, *F* (5, 12) = 7.190). The analysis used is one-way ANOVA followed by Bonferroni *post hoc* test, *n* = 6 hippocampi/treatment. The experiment was repeated three times where three membranes were stripped and re-probed for the three antibodies. However, the representative westerns shown in the figure were all from the same membrane, stripped and re-probed, with the corresponding β-actin blot shown in **D**.

To evaluate differences between the effects of the oligomers on synapses using biochemical analyses, we measured the levels of several synaptic markers in the hippocampi of wild-type mice after treatment with the oligomers. The markers included PSD95, a post-synaptic marker; Synaptophysin, a presynaptic marker; and drebrin, a synaptic spines marker. For biochemical analyses, western blot assays (Fig. 5A–C) were performed using synaptosomal fractions of fresh intact hippocampi that were aerated and incubated with each of the oligomers for 1 h on ice. We observed a significant decrease in PSD95 levels with all the oligomers except with the sham when compared with the untreated control (Fig. 5E). However, PSD95 levels decreased significantly only with the repeated blast oligomers treatment when compared with sham treatment among the groups. Synaptophysin levels significantly decreased with all treatments including with sham oligomers in comparison to untreated control. However, the decrease was significant only with the RB-3Wk and the recombinant tau 4R oligomers when compared with sham oligomers treatment (Fig. 5F). Drebrin levels (Fig. 5G) significantly decreased only with the RB-3Wk oligomer when compared with both sham and untreated control. This indicates that the RB-3Wk oligomers might affect the organization and the integrity of the pre- and post-synaptic densities and synaptic spines, thus affecting synaptic function. All data were normalized against the β-actin. An ‘n’ of three hippocampi was used for each group. Western blots were repeated in triplicates.

### Single versus repetitive mTBI brain-derived tau oligomers represent different polymorphisms

**Figure 6:**
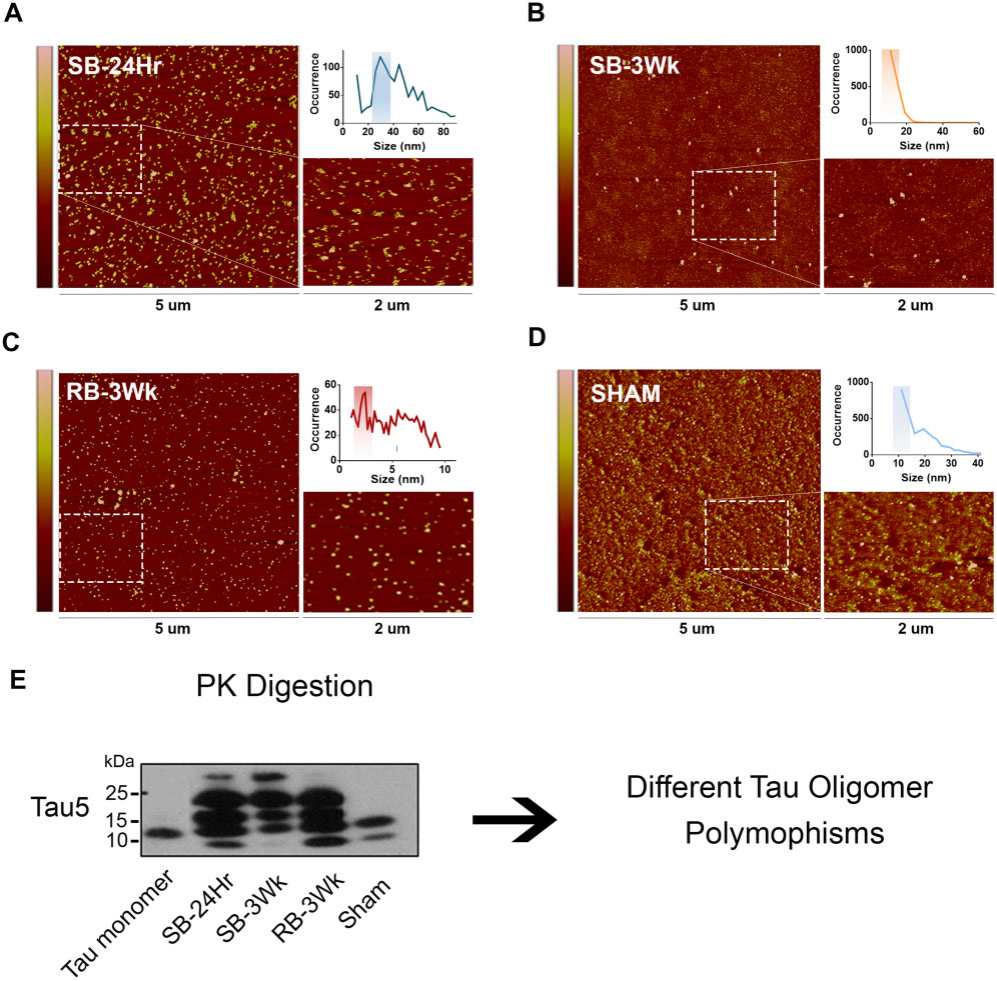
**Tau oligomers derived from brains exposed to single versus repetitive TBI represent different tau polymorphisms.** TBI brain-derived tau oligomers were analysed using AFM. (**A**) SB-24Hr, (**B**) SB-3Wk, (**C**) RB-3Wk and (**D**) Sham oligomers show different oligomeric morphologies and size distribution profiles (shown by the graphical inlets on the top right of each panel). (**E**) Western blot representing the oligomers after PK treatment, probed with Tau5, total tau antibody. The samples show different banding patterns after PK digestion indicating different tau oligomeric polymorphisms.

Results so far suggest that the oligomers isolated from single versus repetitive TBI-exposed brains express different biochemical and functional characteristics. This leads to the question of whether these oligomeric samples represent different polymorphisms. First, we utilized AFM to explore differences between the oligomers in morphology and size. The oligomers appear in different morphologies and express different size distribution profiles. The RB-3Wk oligomer (Fig. 6C) shows the highest occurrence in the 2–4 nm size range. However, the SB-24Hr (Fig. 6A), SB-3Wk (Fig. 6B) and the sham oligomers (Fig. 6D) all occur the most in size ranges above 10 nm.

Different morphological features further suggest that the oligomeric samples represent different polymorphisms. Could they represent different strains? Therefore, we used PK digestion to explore strain differences between the four groups of oligomers (Fig. 6E). The oligomers appear in different patterns and intensities of bands after PK digestion, which indicates differential core protease resistance potentials between the groups. This strongly suggests that the four oligomeric samples represent different strains. Nevertheless, to consider them strains, the seeding abilities of the different groups should be examined.

### Single versus repetitive mTBI brain-derived tau oligomers are potent in seeding and express differential seeding morphologies

**Figure 7:**
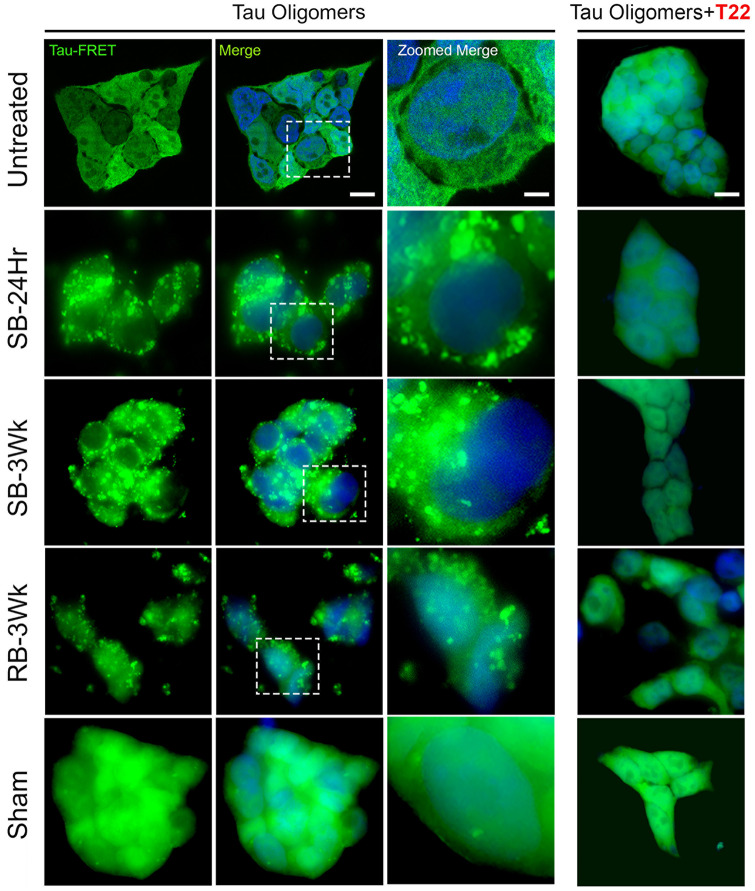
**Tau oligomers derived from brains exposed to single versus repetitive TBI show different seeding patterns and morphologies observed in Tau RD P301S FRET biosensor human cell line.** Tau RD P301S FRET biosensor cells were exposed to a sub-lethal concentration (0.125 µM) of the oligomers alone or oligomers pre-incubated with T22 antibody (1:4 ratio oligomer to antibody) for 48 h in the presence of lipofectamine. All the oligomeric groups showed prominent seeding and unique aggregation patterns except the sham oligomers. Prominent seeding was not observed earlier than 48 h. (Merge scale bar: 10 µm, Zoomed Merge scale bar: 2 µm).

To test the potency of TBI brain-derived tau oligomers in seeding, we utilized a widely used and well-established cell line, the Tau RD P301S FRET Biosensor cells (Holmes *et al.*, 2014; Furman *et al.*, 2015; Kaufman *et al.*, 2016). The cells were exposed to a sub-lethal concentration of TBI brain-derived tau oligomers (0.125 µM) for 48 h in the presence of lipofectamine (Fig. 7) (Toxicity characterization of the 0.125 µM oligomer dose is shown in Supplementary Fig. 2). SB-24Hr, SB-3Wks and RB-3Wks oligomers induced apparent aggregation which confirms their seeding ability when compared with the untreated control. Sham oligomers induced few aggregates that were spotted sparsely distributed without affecting the cellular integrity. An interesting observation was that oligomers from single and repetitive blast injuries expressed different aggregation patterns and distribution in the cell (Holmes *et al.*, 2014; Furman *et al.*, 2015; Kaufman *et al.*, 2016). Combined with the seeding ability clearly shown in Fig. 7, results indicate that the TBI brain-derived tau oligomeric samples might represent different strains.

To confirm that the seeding/aggregation was mediated by tau oligomers, the oligomeric samples were pre-incubated with T22 antibody for 2 h at a ratio of 1:4 (oligomer to antibody). The Tau RD P301S FRET biosensor cells were then treated with the oligomer-T22 complex. Figure 7 shows a complete reversal of seeding/aggregation.

## Discussion

In this study, we investigated the differences between tau oligomers isolated from wild-type mouse brains that were exposed to single and repetitive mTBI to inspect the mechanisms behind the differential risks for neurodegeneration observed after different mTBI frequencies. We found that this differential risk could be attributed to the development of different tau polymorphisms/strains after single versus repetitive mTBI that express differential toxicity, functional impairment and seeding profiles.

Although several studies have introduced and investigated the notion of tau strains, our study is novel in investigating strains after different TBI frequencies. This study is also novel in the systemic approach developed to characterize the differences between tau polymorphisms. In this approach, we used brain-derived immunoprecipitated tau oligomers, unlike other studies that follow a less specific approach of using whole brain homogenates to test tau’s effect on cellular culture or LTP impairment. Moreover, we chose to use tau oligomers isolated from wild-type mice, and not transgenic or tau overexpressing mouse models because the latter do not represent sporadic tauopathies (Narasimhan and Lee, 2017) and, more importantly, TBI-induced pathologies in normal brains. Our results show for the first time that wild-type mouse tau misfolds, forms different polymorphisms/strains that are in turn able to seed human recombinant tau into their initial polymorphism (shown by the success of the FRET tau RD cells seeding assay). Recent studies provide support for the choice of wild-type mouse tau by showing that pathological brain-derived human tau seed mouse endogenous tau and recapitulate human pathology in wild-type non-transgenic model systems (Guo *et al.*, 2016; Narasimhan *et al.*, 2017). This suggests that wild-type mouse tau adopts the form of misfolded tau and propagates it regardless of the sequence of origin. Moreover, considering the difficulty of obtaining mTBI human samples early after injury, the mouse immunoprecipitation approach followed in this article is shown to be an effective method to study tau aggregation and seeding after TBI in a nontransgenic and thus more physiologically relevant system. Whether these tau seeds will recapitulate TBI-induced neurodegeneration pathology *in vivo* is still warranted.

Tau oligomers have been shown to self-propagate, nucleate normal forms of tau in the cell and seed them into a toxic polymorphism *in vivo* (Kaufman *et al.*, 2016). The detection of tau oligomers as early as 24 h post-TBI (Hawkins *et al.*, 2013) and in late stages after TBI is one evidence of tau seeding, propagation and the highly probable link to neurodegeneration (Cruz-Haces *et al.*, 2017). A recent study by Ojo *et al.* (2016) reported an increase in tau oligomers in the brain up to 3 months after repetitive TBI (Ojo *et al.*, 2016). Another study by Kanaan *et al.* (2016) compared tau aggregates in human brains with chronic traumatic encephalopathy (CTE) and Alzheimer’s disease. They found that tau oligomers increase significantly in brains diagnosed with CTE (stages I–IV) and Alzheimer’s disease in comparison to non-demented control brains. Interestingly, the levels of soluble tau oligomers in CTE stage IV and Alzheimer’s disease were very similar (Kanaan *et al.*, 2016). In addition, after TBI, several factors such as haemorrhages, changes in cerebral blood flow, cerebrovascular damage, axonal damage and neuronal hyperexcitability (Johnson *et al.*, 2013; Carron *et al.*, 2016; Jullienne *et al.*, 2016; Mayeux *et al.*, 2017; Salehi *et al.*, 2017) correlate with tau release, increase in phosphorylation, aggregation and reduction in clearance from brain (Iliff *et al.*, 2014). Overall, it has been established that the levels of soluble tau oligomers in late-stage Alzheimer’s disease brains correlate with memory loss. Therefore, similarly, the elevation, spreading and long-term presence of tau oligomers in brains after TBI is most likely linked to neurodegeneration later on. Our study investigated the presence of tau oligomers in the mice brains for up to 3 weeks after injury. Our results add to the mentioned studies above and support the notion that the endogenous tau after TBI can deform, acquire toxicity and seeding abilities that most likely correlate to tau spreading and propagation, and thus neurodegeneration.

Our results do not only suggest a possible correlation of tau oligomers to neurodegeneration, but also the differential PK digestion pattern and FRET seeding pattern observed within the groups strongly indicates that different tau oligomeric strains result from different TBI frequencies. Although farfetched, those findings suggest the hypothesis that different oligomeric strains after mTBI could correspond to the differential risk and form of neurodegeneration.

On a related note, Falcon *et al.* (2019) were able to determine the structure of tau filaments from CTE brains for the first time using cryo-electron microscopy. They showed that CTE tau filaments, like Alzheimer’s disease filaments, consisted of 3R and 4R repeats, however, were distinct from Alzheimer’s disease filaments in several crucial aspects. The CTE filaments had a different ß-helix conformation and a hydrophobic cavity that incorporated a non-protein cofactor. They believed that the sequestration of this cofactor may be the result of the physical head trauma and may affect the pre-filamentous aggregation process leading to distinct conformations from those found in Alzheimer’s disease brains. These CTE tau conformers were also stable enough to propagate and seed differently and, therefore, induced different tauopathies (Falcon *et al.*, 2019). The CTE samples investigated in the Falcon study were obtained from a football player and two boxers that suffered from repetitive TBI. The tau conformers among these three samples were conserved. Therefore, do different levels/frequencies of head trauma led to differential sequestration of protein cofactors and thus the formation of different CTE tau conformers/strains? The mechanisms of tau cofactor sequestration, misfolding and aggregation after TBI are still to be determined.

Investigating the effect of the different oligomeric groups on basal neuronal transmission and LTP in fresh brain slices is an *ex vivo* assay and is the closest to an *in vivo* investigation of the functional characteristics of the oligomeric isolates. It becomes worthy to mention that our results show for the first time that mTBI brain-derived tau oligomers affect basal neuronal transmission. One of the mechanisms through which tau spreads in the brain is through neuronal transmission/firing. Neuronal discharge has been shown to increase after TBI in animal models and humans (Carron *et al.*, 2016). Levels of glutamine and acetylcholine have been shown to drastically increase in the first few hours after injury leading to a state of hyperexcitability. However, this state is followed by a general state of suppression or neuronal inhibition due to neuronal high energy consumption (Barkhoudarian *et al.*, 2011). The elevation in neuronal excitability observed only after treatment with oligomers isolated 3 weeks after repetitive blast could explain the greater LTP impairment and the complete abolishment of paired-pulse facilitation observed. On the other hand, the oligomers isolated 24 h after a single blast showed a decrease in basal neuronal transmission. This could be explained by the ability of the neurons to suppress hyperexcitability and thus suppress neuronal exhaustion with these less-impairing SB-24Hr oligomers before further cellular damage is induced. These findings support again the notion that these oligomeric species are distinct.

Collectively, we demonstrate for the first time that different mTBI frequencies lead to the formation of distinct tau polymorphisms with different toxicity and impairment potentials that could predict the risk for neurodegeneration and dementia after mTBI. This study highlights the importance of identifying neurodegeneration-relevant polymorphisms of tau and developing different therapies specific for each of the toxic oligomeric polymorphisms. This highlights the importance of developing specific immunotherapy intervention approaches that express high specificity and clearance potentials of the toxic tau oligomers before the onset of neurodegeneration. Alongside these studies, it will also be crucial to investigate the effect of the host on tau oligomer formation; for factors like the genetic makeup such as APOE (Nathoo *et al.*, 2003) and TREM2 (Bemiller *et al.*, 2017; Leyns *et al.*, 2017) alleles also affect tau oligomers and thus contribute to determining the risk for neurodegeneration and late-life dementia (Little *et al.*, 2014).

## Supplementary Material

fcz004_Supplementary_Data

## Abbreviations

ACSF: artificial cerebral spinal fluid
AFM: atomic force microscopy
ANOVA: analysis of variance
BBB: blood–brain barrier
CTE: chronic traumatic encephalopathy
ECL: enhanced chemiluminescence
fEPSP: field excitatory post-synaptic potential
FPLC: fast pressure liquid chromatography
FRET: fluorescence resonance energy transfer
HFS: high-frequency stimulation
IP: immunoprecipitation
LDH: lactate dehydrogenase
LTP: long-term potentiation
mTBI: mild traumatic brain injury
OD: optical density
PBS: phosphate-buffered saline
PK: proteinase K
RB-3Wk: repeated blast 3 weeks
SB-24Hr: single blast 24 hours
SB-3Wk: single blast 3 weeks
TBI: traumatic brain injury
TBS-T: tris-buffered saline Tween
TOMA: tau oligomer monoclonal antibody
