# Supplementary material for: Neurotoxic tau oligomers after single versus repetitive mild traumatic brain injury
Source: Brain Commun. 2019 Jun 28;1(1):fcz004. doi: 10.1093/braincomms/fcz004 (PMC6777515; doi:10.1093/braincomms/fcz004)
Supplement: fcz004_Supplementary_Data [file fcz004_supplementary_data.zip › Supplementary Figure Legends.docx]

**Supplementary figures:**

**Supplementary Fig. 1: Characterization of the brain homogenates and original immunoprecipitated samples.** (A) Conventional IP-inputs and IPed TBI brain-derived tau oligomers were probed with Tau 5 antibody. (B) The same blot was performed using a control anti-IgG antibody, which detected faint bands in the input panel but not in the IPed tau oligomers panel. (C) Filter trap assay of amplified IPed TBI brain-derived tau oligomers showing that all samples are detected by C-terminal and N-terminal tau antibodies Tau46 and Tau13, respectively. (D) FPLC chromatogram of TBI brain-derived tau oligomers; The major peaks in SB-24Hr, SB-3Wk, and sham samples correspond to monomers, dimers, and trimers (40-75 kDa and 100-250 kDa). RB-3Wk peaks correspond to dimers and trimers (100-250 kDa) and lacked peak corresponding to monomers.

**Supplementary Fig. 2: Toxicity assay of TBI brain-derived tau oligomers at a sub-lethal dose.** SH-SY5Y cells were exposed to sub-lethal concentration of mTBI brain-derived tau oligomers (0.125 µM). (A) MTT assay results show that the oligomers decrease cellular viability by an average of 30%. No statistically significant differences in cellular viability was observed between the groups at this low concentration. A trend shows that tau4R recombinant oligomers reduced percent cellular viability more that the TBI brain-derived oligomers. (B) Negligible amounts of % cytotoxicity was detected via the LDH assay in all the groups.
