# Supplementary figures and images for: Neurotoxic tau oligomers after single versus repetitive mild traumatic brain injury
Source: Brain Commun. 2019 Jun 28;1(1):fcz004. doi: 10.1093/braincomms/fcz004 (PMC6777515; doi:10.1093/braincomms/fcz004)

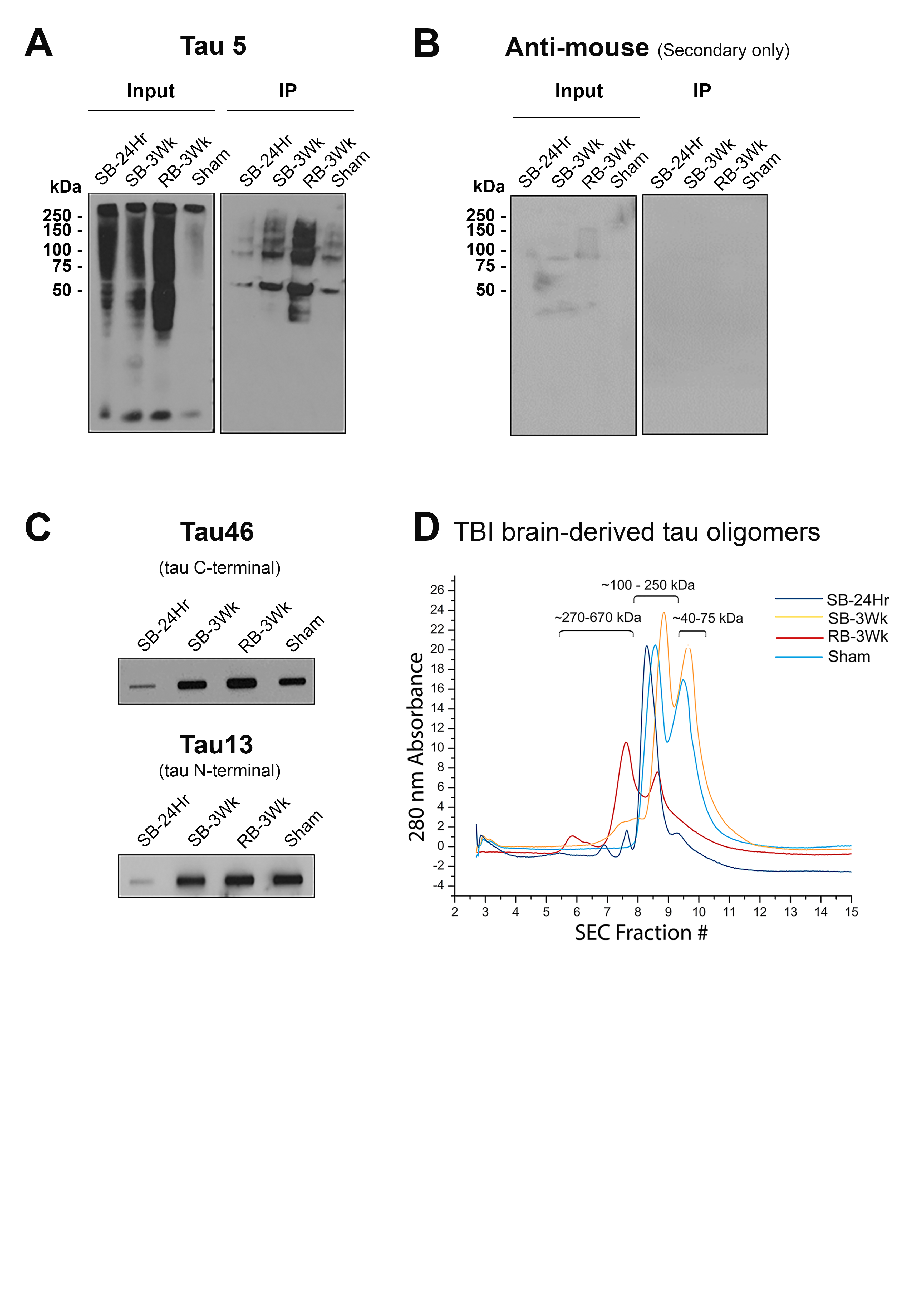

Supplement: fcz004_Supplementary_Data [file fcz004_supplementary_data.zip › Supp fig.1 6001 dpi.tif]
